# Osseous metaplasia of the endometrium associated with infertility: a case report and review of the literature

**DOI:** 10.4076/1752-1947-3-7427

**Published:** 2009-09-10

**Authors:** Julio César Rosa-e-Silva, Ionara Diniz Barcelos, Paula Andrea Navarro, Ana Carolina Japur de Sá Rosa-e-Silva, Antonio Alberto Nogueira, Rui Alberto Ferriani

**Affiliations:** 1 Department of Gynecology and Obstetrics, Faculty of Medicine of Ribeirão Preto, University of São Paulo, São Paulo, Brazil

## Abstract

**Introduction:**

Endometrial ossification is an uncommon disease related to secondary infertility and its etiology and pathogenesis are controversial. More than 80% of reported cases occur after pregnancy.

**Case presentation:**

A 33-year-old Caucasian woman was admitted with a history of secondary infertility and with a regular menstrual cycle. She reported a miscarriage at 12 weeks of gestation 7 years previously and subsequent dilatation and curettage in another medical facility. Vaginal ultrasound was performed and showed an intrauterine structure described as a hyperechogenic image suggesting calcification related to chronic endometritis. Office hysteroscopy revealed a wide endometrial cavity and proliferative endometrium, with a coral-like white plaque 1.5 cm in length on the right horn and posterior wall of the uterus. The lesion was treated by hysteroscopy without complications. Microscopic examination showed endometrial tissue with osseous metaplasia in the stroma. Nine months after the procedure, the patient became pregnant spontaneously.

**Conclusion:**

In our patient, hysteroscopy was effective in the diagnosis and treatment of osseous metaplasia of the endometrium associated with infertility.

## Introduction

Endometrial ossification is an uncommon disease related to secondary infertility and its etiology and pathogenesis are controversial. More than 80% of reported cases occur after pregnancy [1]. The most widely accepted hypothesis is that ossification represents retained fetal bones following spontaneous, missed, incomplete or therapeutic abortion, suggesting endochondral ossification. It can also be related to transformation of mesenchymal tissue to bone in response to inflammation and the reparative process induced by abortion [2]-[4]. A few cases of endometrial ossification occur after abortion at a very early stage of gestation or without a previous history of pregnancy, suggesting a phenomenon of true heterotopia with metaplasia of mature endometrial stromal cells [5]. Osseous metaplasia is rare and can be misdiagnosed. The gold standard for its diagnosis and treatment is hysteroscopy [6].

We present the case of a patient with endometrial ossification associated with secondary infertility after a miscarriage at 12 weeks, suggesting osseous metaplasia. The patient was successfully treated by hysteroscopy. We also present a literature review.

## Case presentation

**Figure 1:**
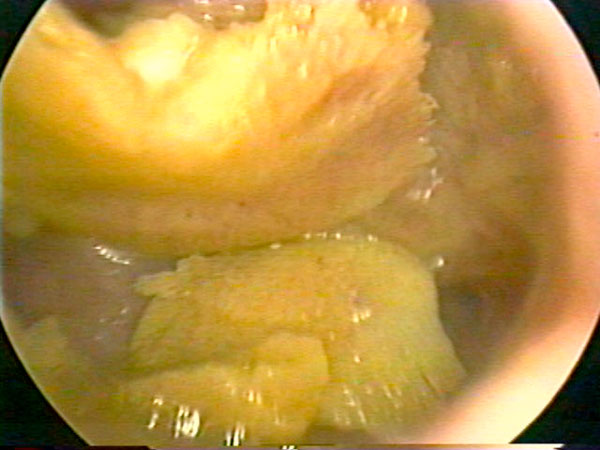
**Hysteroscopic aspect of osseous metaplasia of the endometrium**.

A 33-year-old Caucasian woman was admitted to our outpatient endoscopic unit with a history of secondary infertility and with a regular menstrual cycle. She reported a miscarriage at 12 weeks of gestation, 7 years previously and she subsequent dilatation and curettage (D&C) in another medical facility. No examinations were performed until this admission, when the patient presented complaining of secondary infertility. Vaginal ultrasound was performed and showed an intrauterine structure described as a hyperechogenic image suggesting calcification related to chronic endometritis. Office hysteroscopy revealed a wide endometrial cavity and proliferative endometrium, with a coral-like white plaque, 1.5 cm in length, on the right horn and posterior wall of the uterus (Figure 1). Biopsy (Novak) was performed and pathological findings showed unspecific chronic endometritis with dystrophic calcification. The lesion was treated by hysteroscopy in a second procedure. Diagnostic hysteroscopies were performed using a Hamou I and II Storz endoscope (Karl Storz, Tuttlingen, Germany) with a 30° 5 mm optic system and the uterine cavity was distended with CO_2_. Operative hysteroscopies were performed under spinal block anesthesia, with a 10 mm Storz resectoscope. Briefly, the uterine cavity was distended with a solution of mannitol up to a pressure of 100 mmHg, and the endometrial osseous lesion was identified and completely removed using the monopolar cutting loop under hysteroscopic control. There were no operative complications. Pathological examination showed endometrial tissue with osseous metaplasia in the stroma. Nine months after the procedure, the patient became pregnant spontaneously.

## Discussion

As early as 1884, Virchow attributed the formation of bone in the endometrium to spontaneous differentiation of fibroblasts into osteoblasts [7]. In 1923, Thaler *et al.* linked the presence of this bony tissue to a previous abortion [5]. In 1956, De Brux *et al.* provided the first description of osteogenesis within the genital tract [8].

Pathogenic mechanisms related to the histogenesis of heterotopic bone into the endometrium are controversial. Many theories have been proposed: osseous metaplasia from multipotential stromal cells, usually fibroblasts, which become osteoblasts [7]; continuous and strong endometrial estrogenic stimulation; retention of fetal bones that secondarily promote osteogenesis in the surrounding endometrium [9]; implantation of embryonic parts without pre-existing bone after abortions at an early stage; dystrophic calcification of retained and necrotic tissues, usually after an abortion; chronic endometrial inflammation such as endometritis or pyometra [10]; and metabolic disorders such as hypercalcemia, hypervitaminosis D or hyperphosphatemia. The actual contribution of these pathogenic mechanisms is unknown [11].

Bhatia and Hoshiko reported a case of osseous metaplasia involving both the endometrium and the endocervix [12]. They believed this could be associated with prolonged chronic inflammation and tissue destruction following repeated spontaneous or therapeutic abortions. Fetal bones might have served as a source of calcium for ossification, but this may be valid only for abortions occurring in the second trimester, when ossification of the fetal skeleton has reached a certain level. Otherwise, ectopic bone formation and calcification result from the insult of chronic inflammation or tissue destruction with repeated abortions [13].

In our patient, the endometrial biopsy provided evidence of unspecific chronic inflammation, which has no well-established relationship with infertility. However, according to Marcus *et al.*, this reactive endometritis was probably caused by the presence of the bone fragments which interferes with blastocyst implantation [10]. Also supporting the presence of inflammation in cases of endometrial osseous metaplasia, it has been documented by Lewis *et al.*[14] that the removal of bone fragments from the endometrium in these cases reduced the local concentrations of prostaglandin in 50%.

Melius *et al.* reported two cases of prolonged intrauterine retention of fetal bones following spontaneous abortions 13 years and 14 months before diagnosis [15]. Although this type of entity is different from osseous metaplasia, the histories and symptoms have much in common. The absence of a surrounding tissue reaction and endochondral ossification may differentiate osseous metaplasia from retained fetal tissue. Osseous metaplasia has an endogenous development. In the case reported by Ganem, some of the bone fragments in the endometrium contained marrow [13]. Outside of the bone fragments, the endometrium may occasionally contain foci of calcification.

It is also probable that the concept of a superoxide radical superoxide dismutase system, which plays an important role in endometrial differentiation, may be functional in osseous metaplasia. Chronic post-abortal inflammation due to retained gestational tissues may promote superoxide radical or tumor necrosis factor release from the mononuclear phagocytes. Endometrium deficient in protective superoxide dismutase activity may perhaps present a long lasting insult to the multipotential stromal cells, and this insult may therefore transform these cells into osteoblasts [16].

Reported cases of endometrial ossification frequently have a history of previous pregnancy loss, but most of them do not make any distinction between intrauterine retention of fetal bones and heterotopic bone formation. Among the few reported cases in the literature, the time lag between antecedent abortion and discovery of the endometrial ossification varies between 8 weeks [17] and 14 years [2].

Endometrial ossification may result in secondary infertility, menstrual irregularities, pain or dysmenorrhea [3,12,18].

Ultrasound examination plays a primary role in the diagnosis of patients with osseous metaplasia. The characteristic hyperechogenic pattern is strongly suggestive of osseous tissue within the uterus and should be confirmed by hysteroscopic examination.

In most of the previously reported cases, hysterectomy or curettage D&C have been used as treatment, but only a few patients have been treated by hysteroscopic procedures ([Table 1]).

**Table 1:** Cases report of endometrial osseous metaplasia treated by hysteroscopy

Case	Reference	Case description
1	Rodriguez BD, Adamson GD: **Hysteroscopic treatment of ectopic intrauterine bone. A case report.***J Reprod Med* 1993, **38:**515-520. [4]	Patient with infertility had a diagnosis of endometrial osseous metaplasia, which was removed successfully by hysteroscopy assisted by laparoscopy. She delivered a healthy infant.
2	Marcus S, Bhattacharya J, Williams G, Brinsden P, Hamou J: **Endometrial ossification: a case of secondary infertility. Report of two cases.***Am J Obstet Gynecol* 1994, **170:**1381-1383. [10]	Report of two cases of osseous metaplasia. One patient conceived spontaneously.
3	Bahceci M, Demirel LC: **Osseous metaplasia of the endometrium: a rare cause of infertility and its hysteroscopic management.***Hum Reprod* 1996, **11:**2537–-2539. [20]	Case report of a patient with secondary infertility after an abortion 12 years previously. Two months after resection of the osseous lesion the patient conceived spontaneously.
4	Coccia ME, Becattini C, Bracco GL, Scarselli G: **Ultrasound-guided hysteroscopic management of endometrial osseous metaplasia.***Ultrasound Obstet Gynecol* 1996, **8:**134-136. [19]	Case report of a patient with metrorrhagia and leucorrhea, with a previous normal delivery and a previous 25-week gestation loss. Osseous metaplasia was diagnosed by hysteroscopy and resected. Patient became asymptomatic after the procedure.
5	Torné A, Jou P, Pagano R, Sanchez I, Ordi J, Vanrell JA: **Endometrial ossification successfully treated by hysteroscopic resection.***Eur J Obstet Gynecol Reprod Biol* 1996, **66:**75-77. [11]	Case report of a patient with dysmenorrhea, with two previous voluntary abortions and a diagnosis of endometrial osseous metaplasia, which was treated by hysteroscopic removal of the lesion, with complete resolution of symptoms.
6	García León F, Kably Ambe A: **Osseous metaplasia of the endometrium as a cause of infertility. Hysteroscopic approach.***Ginecol Obstet Mex* 1999, **67:**37-41. [21]	Patient with infertility with hysteroscopic diagnosis of osseous metaplasia, which was resected surgically.
7	Van den Bosch T, Dubin M, Cornelis A: **Favourable pregnancy outcome in a woman with osseous metaplasia of the uterus.***Ultrasound Obstet Gynecol* 2000, **15:**445-447. [22]	Patient with diagnosis of endometrial osseous metaplasia 20 days after a spontaneous delivery. The patient had had an abortion many years earlier.
8	Lainas T, Zorzovilis I, Petsas G, Alexopoulou E, Lainas G, Ioakimidis T: **Osseous metaplasia: case report and review.***Fertil Steril* 2004, **82:**1433-1435. [6]	Case report of endometrial osseous metaplasia associated with secondary infertility. The lesion was removed hysteroscopically using a resectoscope. The patient then had an *in vitro* fertilization (IVF) procedure resulting in the delivery of a healthy infant.
9	Onderoglu LS, Yarali H, Gultekin M, Katlan D: **Endometrial osseous metaplasia: an evolving cause of secondary infertility.***Fertil Steril* 2008, **90:**2013.e9-11. [23]	Patient with secondary infertility after two abortions. Endometrial osseous metaplasia was diagnosed and successfully removed by hysteroscopy.

In patients with extensive osseous metaplasia and bony sheets embedded in the myometrium, satisfactory hysteroscopic removal is difficult. In such cases, the utility of laparoscopic control during the procedure has been reported resulting in greater accuracy and prevention of complications such as uterine perforation [4]. Also, ultrasound-guided hysteroscopy may be an efficient method of minimizing complication risks; nevertheless, it depends on the ability of the ultrasound examiner [19].

## Conclusion

Hysteroscopy has been shown to be effective in the diagnosis and treatment of cases of osseous metaplasia of the endometrium associated with infertility.

## Abbreviations

D&C: dilatation and curettage; IVF: *in vitro* fertilization.

## Consent

Written informed consent was obtained from the patient for publication of this case report and any accompanying images. A copy of the written consent is available for review by the Editor-in-Chief of this journal.

## Competing interests

The authors declare that they have no competing interests.

## Authors' contributions

JCR and IDB made substantial contributions to the design, acquisition of data, literature review and drafting of the manuscript. PAN, ACJSR, RAF and AAN were responsible for the drafting and general revision of this work. All authors have approved the final manuscript.

## References

[B1] Mayer R Knochegnewene im fotalen uterus Z Geburtshilfe Gynekol 1901 46 490 492

[B2] Ceccacci L Clancy G Endometrial ossification: report of an additional case Am J Obstet Gynecol 1981 141 103 104 6791501

[B3] Acharya U Pinion SB Parkin DE Hamilton MPR Osseous metaplasia of the endometrium treated by hysteroscopic resection Br J Obstet Gynaecol 1993 100 391 392 8494844 10.1111/j.1471-0528.1993.tb12988.x

[B4] Rodriguez BD Adamson GD Hysteroscopic treatment of ectopic intrauterine bone. A case report J Reprod Med 1993 38 515 520 8410844

[B5] Thaler H Uberlebendes fotales knorpelgewebe in der uterushohel nach abortus Zentralbl Gynakol 1923 46 1784 1787

[B6] Lainas T Zorzovilis I Petsas G Alexopoulou E Lainas G Ioakimidis T Osseous metaplasia: case report and review Fertil Steril 2004 82 1433 1435 10.1016/j.fertnstert.2004.04.055 15533373

[B7] Virchow R Ueber metaplasia Vichows Arch Abt Pathol Anat 1884 97 410

[B8] De Brux J Palmer R Ayoub-Despois H Les ossification de l'endometre Gynecol Obstet 1956 55 494 504 13405282

[B9] Newton CW III Abel MR Iatrogenic fetal implants Obstet Gynecol 1972 40 686 691 5083218

[B10] Marcus SF Bhattacharya J Williams G Brinsden P Hamou J Endometrial ossification: a case of secondary infertility. Report of two cases Am J Obstet Gynecol 1994 170 1381 1383 8178874 10.1016/s0002-9378(94)70164-4

[B11] Torné A Jou P Pagano R Sanchez I Ordi J Vanrell JÁ Endometrial ossification successfully treated by hysteroscopic resection Eur J Obstet Gynecol Reprod Biol 1996 66 75 77 10.1016/0301-2115(95)02376-3 8735764

[B12] Bhatia NN Hoshiko MG Uterine osseous metaplasia Obstet Gynecol 1982 60 256 259 6818502

[B13] Ganem KJ Parsons L Friedell GH Endometrial ossification Am J Obstet Gynecol 1962 83 1592 1594 13896629 10.1016/0002-9378(62)90175-8

[B14] Lewis V Khan-Dawood F King M Beckham C Dawood MY Retention of intrauterine fetal bone increases menstrual prostaglandins Obstet Gynecol 1990 75 561 563 2406670

[B15] Melius FA Julian TM Nagel TC Prolonged retention of intrauterine bones Obstet Gynecol 1991 78 919 921 1923229

[B16] Sugino N Shimamura K Takiguchi S Tamura H Ono M Nakata M Nakamura Y Ogino K Uda T Kato H Changes in activity of superoxide dismutase in the human endometrium throughout the menstrual cycle and in early pregnancy Hum Reprod 1996 11 1073 1078 8671393 10.1093/oxfordjournals.humrep.a019299

[B17] Waxman M Moussouris HF Endometrial ossification following an abortion Am J Obstet Gynecol 1978 130 587 588 415613 10.1016/0002-9378(78)90083-2

[B18] Hsu C Endometrial ossification Br J Obstet Gynaecol 1975 82 836 839 811249 10.1111/j.1471-0528.1975.tb00584.x

[B19] Coccia ME Becattini C Bracco GL Scarselli G Ultrasound-guided hysteroscopic management of endometrial osseous metaplasia Ultrasound Obstet Gynecol 1996 8 134 136 10.1046/j.1469-0705.1996.08020134.x 8883319

[B20] Bahceci M Demirel LC Osseous metaplasia of the endometrium: a rare cause of infertility and its hysteroscopic management Hum Reprod 1996 11 2537 2539 8981150 10.1093/oxfordjournals.humrep.a019154

[B21] García León F Kably Ambe A Osseous metaplasia of the endometrium as a cause of infertility. Hysteroscopic approach Ginecol Obstet Mex 1999 67 37 41 10085608

[B22] Van den Bosch T Dubin M Cornelis A Favourable pregnancy outcome in a woman with osseous metaplasia of the uterus Ultrasound Obstet Gynecol 2000 15 445 447 10.1046/j.1469-0705.2000.00105.x 10976492

[B23] Onderoglu LS Yarali H Gultekin M Katlan D Endometrial osseous metaplasia: an evolving cause of secondary infertility Fertil Steril 2008 90 2013.e9 11 10.1016/j.fertnstert.2008.01.001 18325514

